# Biodistribution and inflammatory profiles of novel penton and hexon double-mutant serotype 5 adenoviruses

**DOI:** 10.1016/j.jconrel.2012.05.025

**Published:** 2012-12-28

**Authors:** Angela C. Bradshaw, Lynda Coughlan, Ashley M. Miller, Raul Alba, Nico van Rooijen, Stuart A. Nicklin, Andrew H. Baker

**Affiliations:** a British Heart Foundation Glasgow Cardiovascular Research Centre, Institute of Cardiovascular and Medical Sciences, University of Glasgow, 126 University Place, Glasgow G12 8TA, UK; b Glasgow Biomedical Research Centre, Institute of Infection, Immunity and Inflammation, University of Glasgow, 120 University Place, Glasgow G12 8TA, UK; c Nanotherapix S.L, Parc Empresarial Can Sant Joan, Avda. De la Generalitat, 152-158, Barcelona, Spain; d Department of Molecular Cell Biology, Vrije Universiteit Medical Center (VUMC), Amsterdam, The Netherlands

**Keywords:** Gene delivery, Adenovirus, Biodistribution, Inflammatory profile, Spleen

## Abstract

The use of adenovirus serotype 5 (Ad5) vectors in the clinical setting is severely hampered by the profound liver tropism observed after intravascular delivery coupled with the pronounced inflammatory and innate immune response elicited by these vectors. Liver transduction by circulating Ad5 virions is mediated by a high-affinity interaction between the capsid hexon protein and blood coagulation factor X (FX), whilst penton–α_v_integrin interactions are thought to contribute to the induction of anti-Ad5 inflammatory and innate immune responses. To overcome these limitations, we sought to develop and characterise for the first time novel Ad5 vectors possessing mutations ablating both hexon:FX and penton:integrin interactions. As expected, intravascular administration of the FX binding-ablated Ad5HVR5*HVR7*E451Q vector (AdT*) resulted in significantly reduced liver transduction in vivo compared to Ad5. In macrophage-depleted mice, increased spleen uptake of AdT* was accompanied by an elevation in the levels of several inflammatory mediators. However ablation of the penton RGD motif in the AdT* vector background (AdT*RGE) resulted in a significant 5-fold reduction in spleen uptake and attenuated the antiviral inflammatory response. A reduction in spleen uptake and inflammatory activation was also observed in animals after intravascular administration of Ad5RGE compared to the parental Ad5 vector, with reduced co-localisation of the viral beta-galactosidase transgene with MAdCAM-1 + sinus-lining endothelial cells. Our detailed assessment of these novel adenoviruses indicates that penton base RGE mutation in combination with FX binding-ablation may be a viable strategy to attenuate the undesired liver uptake and pro-inflammatory responses to Ad5 vectors after intravascular delivery.

## Graphical abstract

**Figure:**
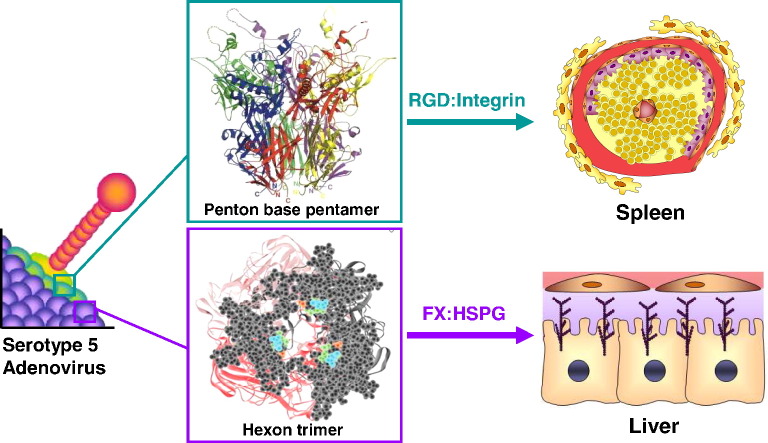

## 1 Introduction

Adenoviruses (Ad) are non-enveloped 70–90 nm DNA viruses that commonly cause a variety of mild illnesses, including gastroenteritis, conjunctivitis and cystitis [1]. Greater than 56 different human Ad serotypes have been identified to date and are classified into species A–G [2]. Ad5-based vectors are relatively easy to manipulate, are able to efficiently transduce a wide variety of dividing and quiescent cell types and it is possible to manufacture clinical grade virus to high titers. Consequently, a significant proportion of gene therapy clinical trials are performed using Ad5-based vectors, which are also frequently used for experimental applications in the laboratory setting [3].

The three major components of the icosahedral Ad5 capsid are the trimeric hexon, pentameric penton base and trimeric fibre proteins. Detailed studies have shown that each capsid component participates in distinct aspects of the interface between Ad5 virions and the cell surface. Early in vitro evidence demonstrated that cell tethering by Ad5 is mediated by an interaction between the fibre knob domain with the coxsackie and adenovirus receptor (CAR; [4]). Subsequent binding of α_v_β_3_/α_v_β_5_ integrins to the exposed arginine–glycine–aspartic acid (RGD) motif in the Ad5 penton base promotes integrin clustering, resulting in the activation of downstream signalling pathways and the induction of cytoskeletal changes required for efficient virus internalisation and endocytosis ([5,6], [7],[8]). In vivo, however, several studies have shown that neither CAR nor α_v_ integrin interactions contribute to the profound liver transduction observed after intravascular (*iv*) delivery of Ad5 in rodents and nonhuman primates [2,9–11]. Indeed, the in vivo localisation of CAR in polarised cells to intercellular tight junctions renders CAR largely inaccessible to circulating Ad5 virions [12,13]. It is now well established that hepatocyte transduction following *iv* delivery is mediated by a high-affinity interaction between the Ad5 hexon protein and circulating blood coagulation factor X (FX), which ‘bridges’ the adenovirus capsid to heparan sulphate proteoglycans (HSPGs) at the cell surface [14–16]. Furthermore, it has been shown that the FX-mediated interaction with highly-sulphated liver HSPGs involves several positively-charged residues in the FX serine protease domain, whilst hypervariable (HVR) regions in the Ad hexon protein bind the FX gamma-carboxylglutamic acid (Gla) domain [16–19]. Based on this knowledge, concerted efforts to generate an optimised Ad5 vector for gene therapy applications via this route of administration have been focused on ablating the nanomolar-affinity FX:Ad5 hexon interaction. Such studies have yielded a series of novel Ad5 vectors possessing mutations within the hexon HVR regions responsible for FX binding, which show markedly reduced liver uptake after *iv* administration [15,17,20,21]. However, we have previously shown that the liver-detargeted, FX binding-ablated Ad5 vector Ad5-HVR5*7*E451Q (hereafter referred to as AdT*), resulted in increased spleen uptake accompanied with moderate increases in the inflammatory cytokines interleukin-12 (IL-12), monokine-induced by interferon gamma (MIG) and 10 kDa interferon gamma-induced protein (IP-10) [22]. The innate immune response to circulating Ad5 virions represents a significant hurdle facing the clinical application of these vectors [23]. The unfortunate death of a phase I clinical trial participant several days after intra-arterial infusion of an Ad5-based vector in 1999 further serves to highlight the importance of developing vectors with limited off-target interactions and improved safety in vivo.

Systemic delivery of Ad5 in mice elicits a marked proinflammatory and cellular immune response both to the viral particle itself and to its expressed genes [24–28]. Studies indicate that recognition of viral DNA by immune sensors such as the NLRP3 inflammasome in circulating monocytes and tissue macrophages plays an important role in the induction of a proinflammatory response to Ad5 [29]. Interestingly, it has also been shown that the Ad5 capsid proteins, specifically the penton base, may also contribute to the induction of this proinflammatory response by promoting macrophage and dendritic cell activation in an RGD integrin-dependent manner [24,30,31]. This observation indicates that mutating the penton base of FX binding-ablated Ad5 vectors to prevent capsid interactions with RGD integrins may potentially attenuate the proinflammatory response to these liver-detargeted Ad5 vectors after *iv* administration. Mutation of the penton base RGD motif in a FX binding-ablated vector would also provide further mechanistic evidence for the involvement of penton base-integrin interactions in spleen uptake after intravascular delivery of AdT*. In this study, we have generated novel Ad single- and double-mutant vectors containing an RGD-ablating point mutation in the penton base of control Ad5 or FX binding-ablated AdT* vectors. We have assessed the effect of these modifications on cell attachment and transduction in vitro and have performed detailed in vivo characterisation of the mutant adenoviruses after *iv* delivery in macrophage-depleted and control mice.

## 2 Materials and methods

### 2.1 Cell lines

The growth requirements of cultured human cell lines HEK293, SKOV3 and A549 have previously been described in detail [18].

### 2.2 Adenoviral vector construction

**Fig. 1:**
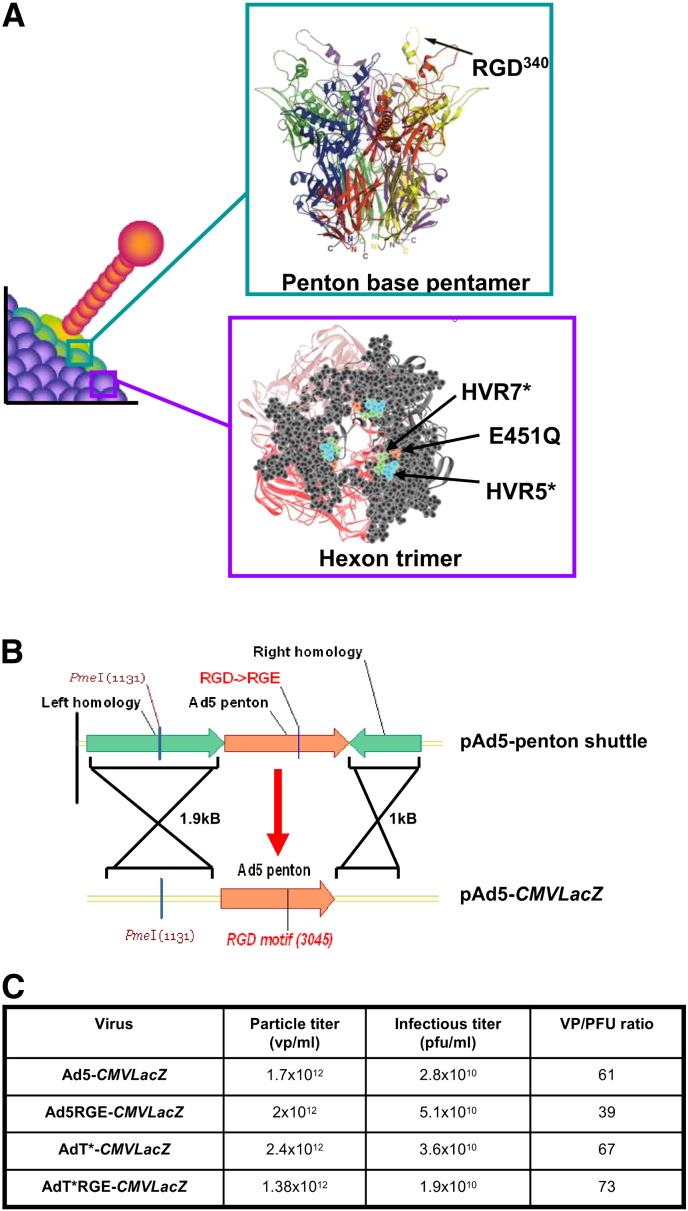
Generation of RGE-mutant Ads. A) Illustration depicting Ad5 vertex, with ribbon diagrams of penton base pentamer and hexon trimer. B) Schematic illustrating homologous recombination of RGE-mutated Ad5 penton base shuttle into the pAd5/AdT*-CMVLacZ backbone. C) Virus particle titers, infectious titers and VP/PFU ratios for all viruses produced.

All viruses are based on the AdEasy, E1/E3-deleted human serotype 5 adenovirus (Ad5), which encodes a CMV-*LacZ* expression cassette (Strategene, Leicester, UK) (Fig. 1A). Construction of the Ad5-HVR5*7*E451Q genome (AdT*) has been described previously [17]. A shuttle vector for homologous recombination within the Ad5 penton base region was generated by amplifying a 4.6 kB fragment containing the penton base sequence, flanked by 1.9 kB (left) and 1 kB (right) regions of homology from the Ad5 genome (For: 5′ ATATGACGAGGACGATGAGTACG, Rev: 5′ CGCCGTACACCTCATCATACAC) (Fig. 1B). An aspartic acid (D) to glutamic acid (E) mutation (5′CGCGGCGAC 3′ -> 5′CGCGGCGAA 3′) was introduced within the penton base RGD motif sequence by site-directed mutagenesis of the 1.3 kB *SexA1-AscI* fragment of the penton base shuttle vector, using the Strataclone blunt cloning kit (Stratagene, Leicester, UK) and the primers For:5′ ATTCGCGGCGAAACCTTTGCCTG and Rev: 5′ GGCAAAGGTTTCGCCGCGAATGG. The *Sex*A1*-Asc*I RGD-mutated fragment was then reintroduced into the penton shuttle plasmid by subcloning and final constructs sequenced and verified using a 3730 DNA analyzer (Applied Biosystems, Warrington, UK). The RGE-mutant penton base shuttle plasmid and the Ad5-CMVLacZ or AdT* genome-containing plasmids were then linearized using *Eco*RI and *Pme*I respectively and homologous recombination performed in BJ5183 electroporation-competent cells according to manufacturer's instructions (Stratagene, Leicester, UK). Recombinant adenovirus genomes were verified by sequencing.

### 2.3 Vector amplification

*Pac*I-digested viral genomes were transfected into HEK293 cells in a 6-well plate using Lipofectamine2000 according to manufacturer's instructions (Invitrogen, Paisley, UK). Cells and media were recovered 5–10 days post-transfection after viral plaques were observed and amplified viruses sequenced (Applied Biosystems, Warrington, UK). Rescued viruses were propagated in HEK293 cells as previously described and purified by double CsCl gradient centrifugation. Viral particles were determined by micro bicinchoninic-acid assay (Perbio Science, Cramlington, UK) according to manufacturer's instructions, using the formula 1 μg protein = 4 × 10^9^ vp [32]. End-point dilution assays were performed to quantify plaque-forming units (PFU)/ml. To verify capsid composition, SDS-polyacrylamide gels were loaded with 5 × 10^10^ vp of each adenovirus and stained using the PageSilver silver staining kit according to manufacturer's instructions (Roche, Welwyn Garden City, UK).

### 2.4 Analysis of Ad cell binding in vitro

SKOV3 or A549 cells were plated in 24-well plates at 1 × 10^5^ cells/well 24 h prior to assay. Cells were gently washed in PBS then incubated in 500 μl serum-free DMEM (SF DMEM) on ice for 30 min. 1 × 10^3^ vp/cell of Ad5, Ad5RGE, AdT* or AdT*RGE in ice-cold SF DMEM was then added to cells, which were incubated for a further 1 h on ice in the presence or absence of FX at a concentration of 10 μg/ml (Cambridge Bioscience, Cambridge, UK). Cells were then washed twice with PBS and pelleted by centrifugation at 8000 × *g*. After lysing cells by freeze-thawing, total cellular DNA was extracted using the QiaQuick DNeasy extraction kit according to the manufacturer's instructions (Qiagen, Crawley, UK). Vector genomes were detected in 100 ng total DNA by SYBR green quantitative polymerase reaction (qPCR) on an ABI Prism 7700 sequence detection system (Applied Biosystems, Warrington, UK) using primers directed against an unmutated portion of the Ad5 hexon gene (For 5′-CGCGGTGCGGCTGGTG-3′and Rev 5′-TGGCGCATCCCATTCTCC-3′). Data are expressed as vp/100 ng DNA.

### 2.5 Analysis of Ad transduction in vitro

SKOV3 or A549 cells were plated out in 96-well plates at 2 × 10^4^ cells/well 24 h prior to infection. Cells were gently washed with PBS then incubated with 1 × 10^3^ vp/cell Ad5, AdT*, Ad5RGE or AdT*RGE in 100 μl SFDMEM for 3 h at 37 °C, in the presence or absence of FX at a concentration of 10 μg/ml (Cambridge Bioscience, Cambridge, UK). Infection media were removed from cells, which were washed gently with PBS prior to addition of 200 μl full medium and incubation at 37 °C for 48 h. Cells were harvested by lysing in 100 μl 0.2% Triton-X-100/PBS. β-galactosidase was quantified using Tropix Galacto-light Plus (Applied Biosystems, Warrington, UK) and visualised by fixing cells in 2% paraformaldehyde for 10 min at room temperature followed by staining in 5-bromo-4-chloro-3-indolyl-ß-d-galactosidase (X-Gal) staining solution for 3 h at 37 °C [0.1 M pH7.3 phosphate buffer, 2 mM MgCl_2_, 5 mM K_3_F_3_(CN)_6_, 5 mM K_4_Fe(CN_6_)_6_, and 1 mg/ml X-Gal]. β-galactosidase activity was quantitated using a Wallac VICTOR2 (PerkinElmer Life and Analytical Sciences, Boston MA) and normalised to total protein concentrations. Protein concentrations were measured by BCA as per manufacturer's instructions. Data are expressed as Relative Light Units (RLU)/mg protein.

### 2.6 Adenovirus delivery in vivo

All animal experiments were approved by the UK Home Office. Male MF1 outbred mice (Harlan, UK) aged between 8 and 10 weeks (weight approx. 35 g) and housed in secure barrier facilities were used for all in vivo experiments. Macrophage depletion was carried out by *iv* administration of 200 μl clodronate-encapsulated liposomes (clodronate liposomes; www.clodronateliposomes.org) 48 h prior to Ad administration. For in vivo transduction studies, 1 × 10^11^ vp Ad was administered by *iv* injection 48 h after PBS (− CD) or macrophage-depletion (+ CD). Mice were sacrificed and perfused with PBS 48 h post-injection. β-galactosidase transgene expression in tissue was assayed using a β-galactosidase enzyme-linked immunosorbent assay (ELISA) kit (Roche, Welwyn Garden City, UK) according to manufacturer's instructions, and normalised to total protein content assayed by BCA as described previously. Liver lobes and spleens that had been fixed in 2% paraformaldehyde (PFA) for 16 h at 4 °C were stained for β-galactosidase activity in X-Gal staining solution (described above) at 37 °C overnight.

### 2.7 Immunohistochemistry in liver and spleen

Tissues were embedded in Optimal Cutting Temperature (OCT) medium-containing cryomolds (Tissue-Tek, U.S.A.) and frozen at − 80 °C immediately after necropsy. To assess the effect of macrophage depletion on defined cell populations, spleen (6 μm) or liver (4 μm) sections from untreated (− CD) or clodronate liposome-treated (+ CD) mice were analysed by immunohistochemistry using antibodies directed against Kupffer cell marker F4/80 and spleen cell markers MARCO, Moma-1 (also known as CD169), B220, MadCAM-1 and ER-TR7, performed exactly as described previously [22]. Co-localisation of viral transgene expression (β-galactosidase) was assessed by staining tissue sections with a rabbit polyclonal anti-β-galactosidase antibody (MP Biomedicals LLC) in combination with F4/80 (liver Kupffer cells), the endothelial marker CD31 (liver), MARCO, Moma-1, B220, MAdCAM-1 or ER-TR7, as previously reported [22]. Immunofluorescence images were acquired on an Olympus BX60 fluorescence microscope using CellM image analysis software (Olympus, Southend, UK) or on a Zeiss LSM510 confocal imaging system using LSM image acquisition software (Carl Zeiss, Welwyn Garden City, UK).

### 2.8 Cytokine and chemokine analysis

Ad5, Ad5RGE, AdT*, AdT*RGE (1 × 10^11^vp) was administered by *iv* injection to untreated (− CD) or macrophage-depleted groups (+ CD). Blood was extracted by venesection and collection in capillary tubes 6 h after virus administration. Blood from PBS controls (−/+ CD) was also collected at this timepoint. Serum was prepared by allowing blood to coagulate for 30 min at room temperature then centrifuging at 10,000 × rpm for 15 min. Cytokine and chemokine analysis of 6 h sera was performed using a mouse cytokine 20-plex luminex panel according to manufacturer's instructions (Invitrogen, Paisley, UK), quantifying levels of basic fibroblast growth factor (bFGF), granulocyte macrophage colony-stimulating factor (GM-CSF), interferon-gamma (IFN-γ), interleukin (IL) 1α, IL-1ß, IL-2, IL-4, IL-5, IL-6, IL-10, IL-12(p40/p70), IL-13, IL-17, IFN-induced protein (IP10), keratinocyte-derived cytokine (KC), monocyte chemoattractant protein (MCP-1), monokine induced by gamma interferon (MIG), macrophage inflammatory protein-1alpha (MIP-1α), tumour necrosis factor-alpha (TNF-α) and vascular endothelial growth factor (VEGF). Data were analysed using Bio-Plex manager software with 5PL curve fitting. Values are presented as pg/ml after correction for the background levels from animals in the PBS groups (+/− CD).

## 3 Results

Two key concerns limiting the clinical application of Ad5 vectors for gene therapy are the profound liver uptake and acute inflammatory activation observed after intravascular delivery. We and others have previously shown that mutation of the HVR regions within the hexon protein results in ablation of Ad5 binding to FX, significantly attenuating hepatocyte transduction [15–17,22]. However, FX binding-ablated Ad5 vectors are hampered by increased spleen uptake after IV administration in macrophage-depleted animals, accompanied by a moderate increase in circulating inflammatory cytokine levels compared with the unmodified Ad5 vector [17]. The underlying mechanism has yet to be elucidated, although there is evidence to suggest that interactions between the penton base RGD motif and α_v_ integrins may be involved [22,24,30]. Thus the goal of the present study was to generate and perform detailed in vitro and in vivo characterisation of novel single and dual mutant Ad5 vectors containing penton base RGE mutations and hexon mutations ablating FX binding.

### 3.1 Construction and characterisation of Ad5RGE and AdT*RGE vectors

Two novel Ad5 vectors based on Ad5 and the FX binding-ablated Ad5 vector AdT* were constructed to assess the role of the penton RGD > RGE motif mutation alone and in combination with FX binding-ablation.

Ad5RGE and AdT*RGE were generated by homologous recombination of a shuttle plasmid containing an RGE-mutated penton base into the Ad5 and AdT* adenovirus backbones respectively, as described in the Materials and methods (Fig. 1A + B). To characterise capsid protein content of mutant and parental vectors, 5 × 10^10^ virus particles of each vector were separated by SDS-PAGE and analysed by silver stain (Fig. S1). Silver stain profiles of all viruses were similar (Fig. S1), indicating that the modifications had not disrupted the structural integrity of the particles. End-point dilution assays to quantify plaque-forming units (PFU) were also performed and VP/PFU ratios calculated (Fig. 1C). Importantly, VP/PFU ratios for all viruses were in the same range, allowing us to directly compare the in vitro and in vivo characteristics of the four vectors.

### 3.2 Similar binding and transduction of human cell lines by mutant Ads

**Fig. 2:**
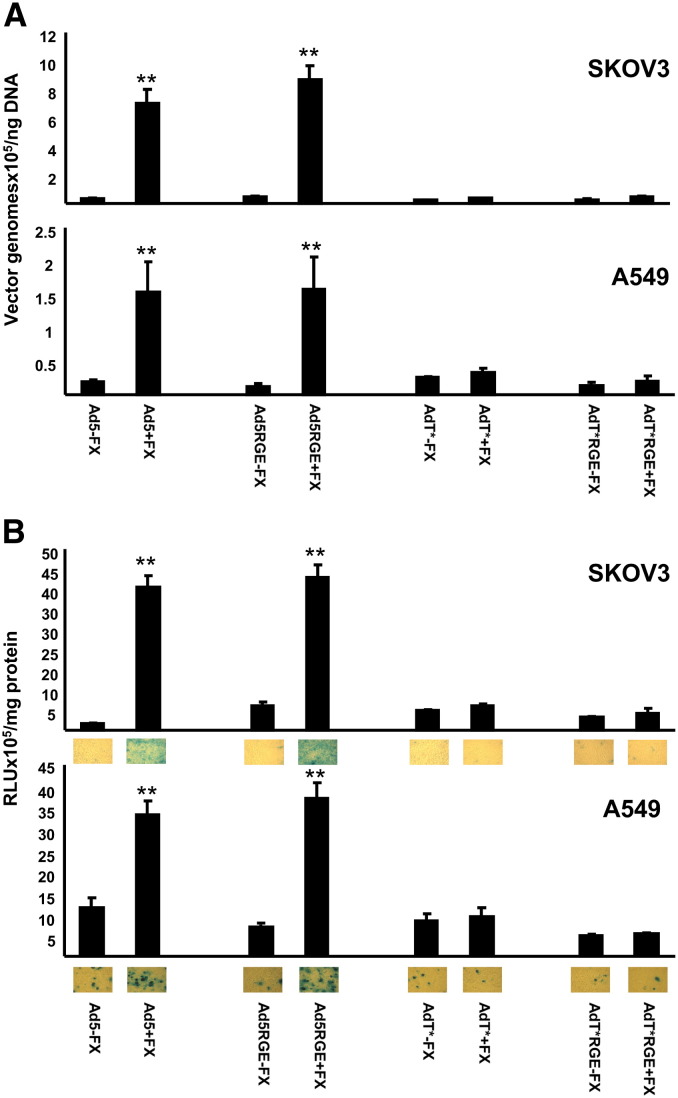
In vitro cell binding and transduction of RGE-mutant Ads. A) Binding of 1000 vp/cell of Ad5, Ad5RGE, AdT* and AdT*RGE to SKOV3 human ovarian carcinoma or A549 human lung carcinoma cells at 4 °C in the presence or absence of a physiological concentration of FX (10 μg/ml). B) Transduction of SKOV3 or A549 cells by Ad5, Ad5RGE, AdT* or AdT*RGE in the presence or absence of 10 μg/ml FX after exposure to cells for 3 h at 37 °C. Cell transduction was assessed by β-galactosidase assay and lacZ staining 48 h post-infection. n = 5, ** = p < 0.01 compared to –FX control.

To assess whether mutation of the penton RGD motif affects in vitro binding and transduction of unmodified or FX binding-ablated Ad, cell attachment and transduction assays were performed in SKOV3 ovarian carcinoma or A549 lung carcinoma cells in the presence or absence of physiological concentrations of FX. Unlike SKOV3 cells, A549 cells express high levels of the primary Ad5 attachment receptor, CAR [33,34]. A significant increase in binding (Fig. 2A) and transduction (Fig. 2B) of both SKOV3 and A549 cells was observed with Ad5 and Ad5RGE viruses in the presence of FX (p > 0.01), consistent with previous studies showing that penton base:integrin interactions are not required for FX-mediated transduction [18,22]. As expected, no FX-mediated increase in binding (Fig. 2A) and transduction (Fig. 2B) of either cell type was observed with AdT* or AdT*RGE. Interestingly, all RGE-mutant viruses retained the ability to transduce both cell types at relatively low levels in the absence of FX, indicating that alternate internalisation pathways may mediate uptake of Ad5 under these conditions.

### 3.3 Modified biodistribution of RGE-mutant Ads after *iv* delivery

**Fig. 3:**
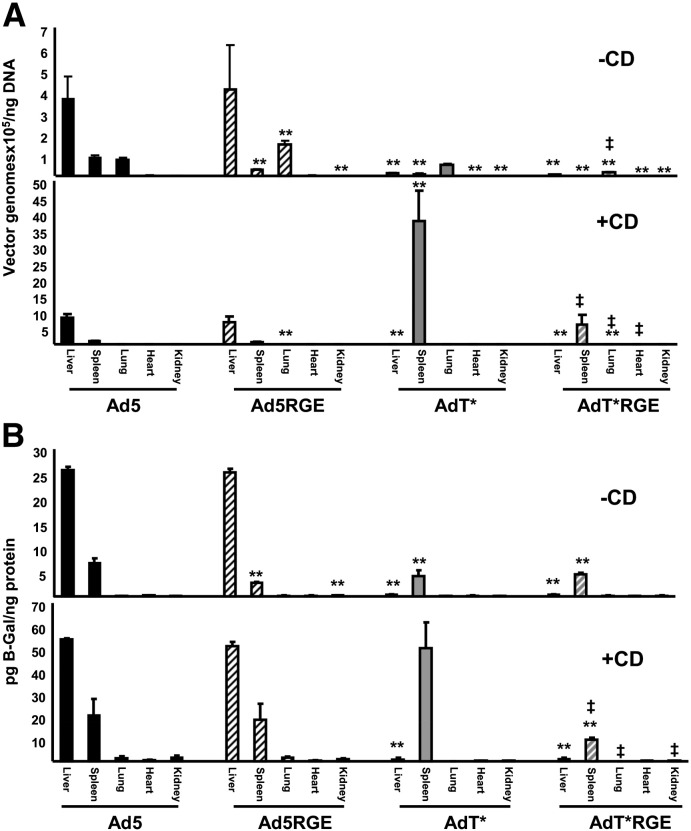
Viral genome content and transduction profiles of tissues from mice injected with parental and RGE-mutant Ads. Viral genome content was assessed by qPCR analysis of DNA extracted from tissues, using primers specific to an unmutated portion of the adenovirus hexon gene. β-galactosidase expression was quantified by ELISA and normalised to total protein content assayed by BCA. A) Viral genome content of the liver, spleen, lung, heart and kidneys from non macrophage-depleted (−CD) and macrophage-depleted (+CD) mice 48 h after *iv* administration of 1 x 10^11^ vp of Ad5, Ad5RGE, AdT* or AdT*RGE. B) β-galactosidase expression in the liver, spleen, lung, heart and kidneys from non macrophage-depleted (−CD) and macrophage-depleted (+CD) mice 48 h after *iv* administration of 1 x 10^11^ vp of Ad5, Ad5RGE, AdT* or AdT*RGE. n=5/group, values expressed as mean +/− SEM. * = p < 0.05, ** = p < 0.01 compared to Ad5. † = p < 0.05, ‡<=<p < 0.01 compared to parental vector (AdT*).

**Fig. 4:**
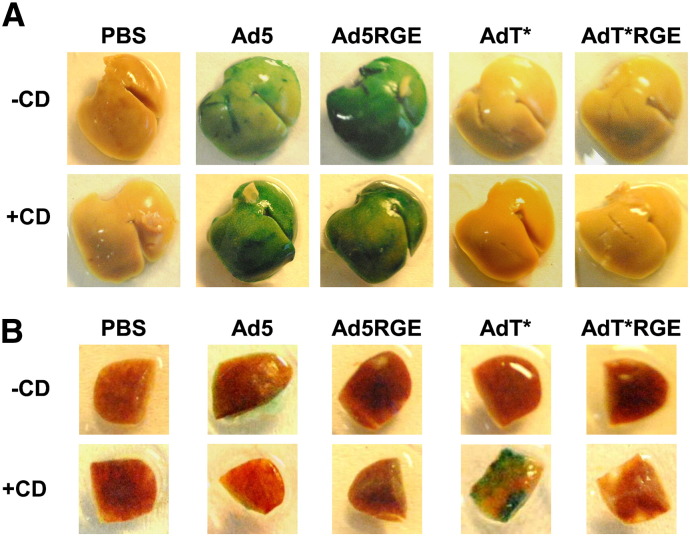
β-Galactosidase staining of livers and spleens from mice injected with parental and RGE-mutant adenoviruses. X-Gal staining of the liver lobes (A) and spleens (B) 48 h after intravascular administration of 1 x 10^11^ vp Ad5, Ad5RGE, AdT* or AdT*RGE.

To characterise the effect of the RGE mutation on the in vivo biodistribution of unmodified and FX binding-ablated Ad5 vectors, we studied the effect of high-dose virus administration in macrophage-depleted and non macrophage-depleted mice. The viral genome content (Fig. 3A) of organs 48 h after *iv* administration of 1 × 10^11^ vp of Ad5, Ad5RGE, AdT* or AdT*RGE was quantified, while viral transgene expression (Fig. 3B) was assessed by β-galactosidase ELISA (Fig. 4A and B). In both macrophage-depleted (+ CD) and non-macrophage-depleted mice (− CD), high levels of liver genome accumulation and transduction were observed after *iv* administration of Ad5 and Ad5RGE (Figs. 3A/B and 4), confirming the redundancy of penton base-integrin interactions for liver uptake in MF1 mice. Conversely, FX binding-ablated vectors mediated significantly reduced levels of liver genome accumulation and transduction in both macrophage-depleted and non-macrophage-depleted animals (p < 0.01; Fig. 3). In non macrophage-depleted AdT* and AdT*RGE animals, vector genome accumulation and β-galactosidase expression were primarily localised to the spleen and lung (Fig. 3; − CD). Low levels of lung transduction and genome accumulation were also observed in non-macrophage-depleted Ad5 and Ad5RGE animals (Fig. 3; − CD). Interestingly, a significant, 5-fold reduction in spleen viral genome accumulation and transduction was observed after Ad5RGE administration to non-macrophage-depleted animals compared to the parental Ad5 vector (Fig. 3, − CD; p < 0.05). In the spleen, we observed a similar reduction in viral genome accumulation and transduction in macrophage-depleted AdT*RGE animals compared to animals administered the parental AdT* vector (Fig. 3, + CD; p < 0.01). There was no significant reduction in spleen transduction of macrophage-depleted animals by Ad5RGE compared to the parental Ad5 vector (Figs. 3 and 4; + CD). In macrophage-depleted animals, significantly higher levels of AdT* spleen transduction was observed compared to non-macrophage-depleted AdT* animals, consistent with previous studies (Figs. 3 and 4, p < 0.01). IV administration of Ad vectors to macrophage-depleted mice led to minimal levels of lung, heart and kidney viral genome accumulation and transduction (Fig. 3; + CD).

### 3.4 Absence of hepatocyte transduction after IV delivery of FX binding-ablated Ads

**Fig. 5:**
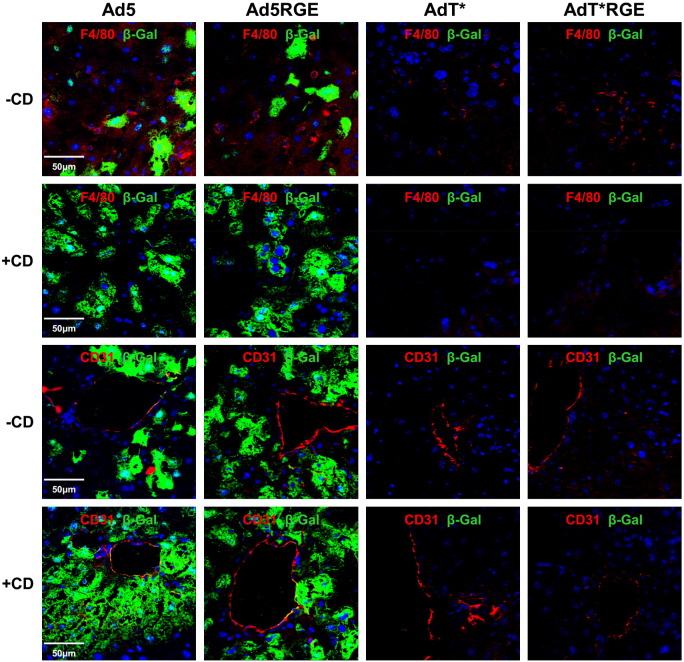
mmunohistochemical analysis of β-galactosidase expression in liver sections. Immunohistochemical analysis of 4 μm frozen liver sections from the control or macrophage-depleted mice injected with 1 x 10^11^ vp Ad5, Ad5RGE, AdT* or AdT*RGE viruses 48 h prior to sacrifice. Sections were stained for β-galactosidase (green) and the macrophage marker F4/80 or the endothelial cell marker CD31. Merged images of liver sections from the control or macrophage-depleted mice injected with 1 x 10^11^ vp of Ad5, Ad5RGE, AdT* or AdT*RGE and stained for s-galactosidase (green) and F4/80 or CD31 (red).

To characterise liver transduction by RGE-mutant Ads after *iv* administration, frozen liver sections from macrophage-depleted and non-macrophage-depleted mice administered 1 × 10^11^ vp of Ad5, Ad5RGE, AdT* or AdT*RGE were analysed by immunohistochemistry. Clodronate liposome pretreatment resulted in significant depletion of F4/80 + liver Kupffer cells (Supplementary Fig. S2; upper panel). Similar, intense staining for β-galactosidase was observed in liver sections from macrophage-depleted (+ CD) and non-macrophage-depleted (− CD) mice administered Ad5 and Ad5RGE, with transgene expression localising primarily to hepatocytes (Fig. 5). A moderate increase in β-galactosidase staining was seen in liver sections from macrophage-depleted (+ CD) Ad5 and Ad5RGE animals compared to non-macrophage-depleted counterparts (Fig. 5). No β-galactosidase expression was observed in liver sections from mice administered AdT* or AdT*RGE (Fig. 5).

### 3.5 Viruses interact with multiple cell types in the spleen

**Fig. 6:**
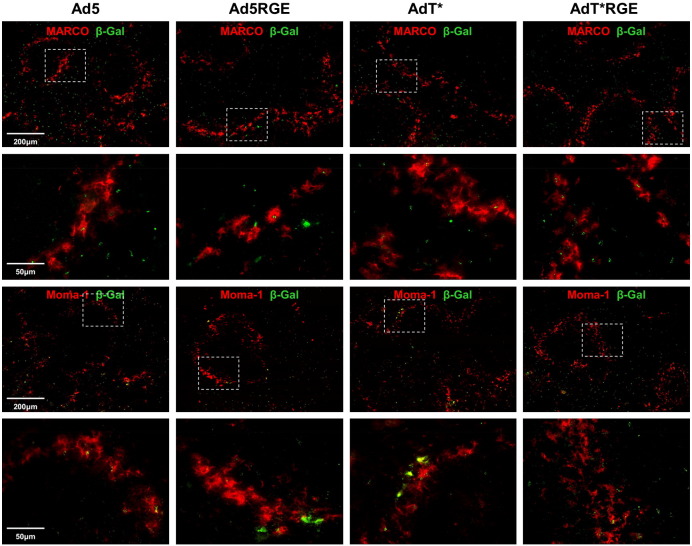
Immunohistochemical analysis of β-galactosidase expression and macrophage markers in the spleens of non-macrophage-depleted animals. Immunohistochemical analysis of 6 μm spleen sections from control (−CD) mice injected *iv* with 1 x 10^11^ vp Ad5, Ad5RGE, AdT* or AdT*RGE 48 h prior to sacrifice. Sections were stained for viral transgene, β-galactosidase (green) and spleen macrophage cell markers MARCO and Moma-1. *Note*: β-galactosidase is abbreviated to β-Gal in figure. Images shown were captured using a 10× or 40× objective and are representative of multiple fields of view. Regions of interest are highlighted by a box and magnified in the corresponding panel below.

**Fig. 7:**
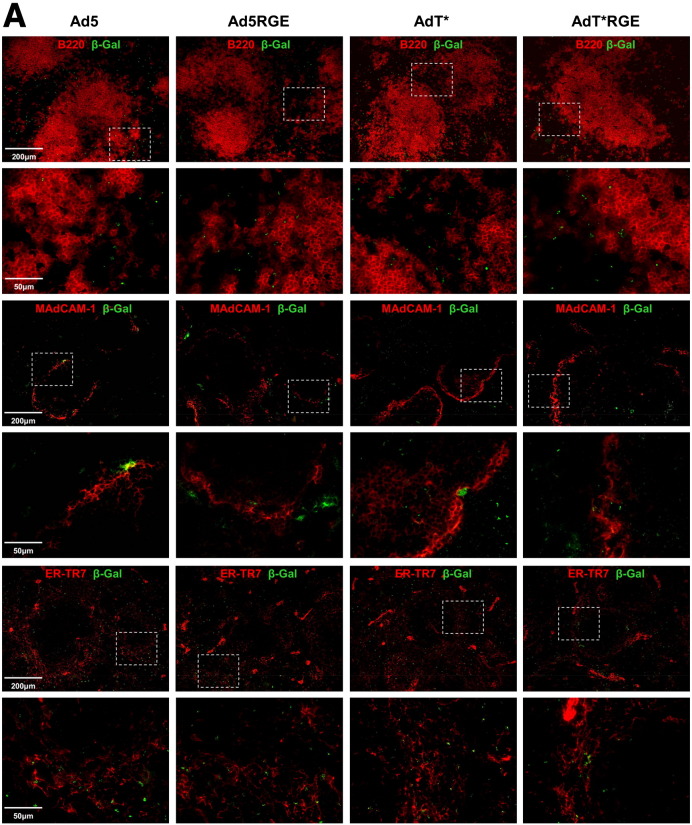

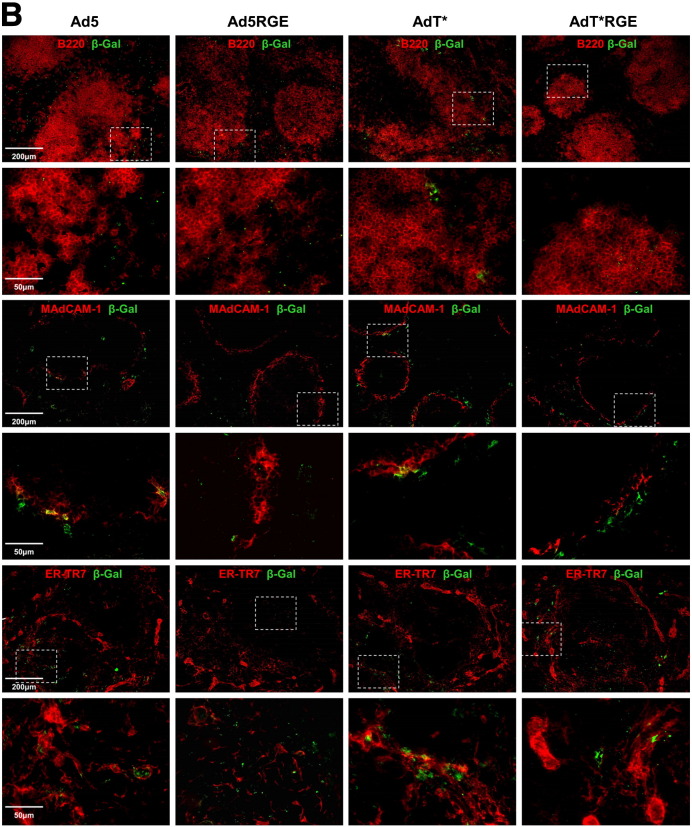
Immunohistochemical analysis of β-galactosidase expression and in the spleens of control (A) and macrophage-depleted animals (B). Immunohistochemical analysis of 6 μm spleen sections from control (−CD) or macrophage-depleted (+CD) mice injected *iv* with 1 x 10^11^ vp Ad5, Ad5RGE, AdT* or AdT*RGE 48 h prior to sacrifice. Sections were stained for viral transgene, β-galactosidase (green) and spleen cell markers B220, MAdCAM-1 or ERTR7 (red). *Note*: β-galactosidase is abbreviated to β-Gal in figure. Images shown were captured using a 10× or 40× objective and are representative of multiple fields of view. Regions of interest are highlighted by a box and magnified in the corresponding panel below.

In order to identify the cell types in the spleen expressing the β-galactosidase transgene 48 h following *iv* delivery of 1 × 10^11^ vp Ad5, Ad5RGE, AdT* or AdT*RGE, we performed co-localisation by immunohistochemistry (Figs. 6 and 7). The distribution of transgene expression in non macrophage-depleted animals (− CD) was largely localised to the marginal zone (MZ), although numerous β-Gal + cells were also detectable within the red pulp (RP) region (Figs. 6 and 7A). The MZ contains specialised macrophages, MARCO + scavenging macrophages and Moma-1 (CD169 +) marginal metallophilic macrophages (MMM), which previously have been shown to trap Ad5 particles at early time-points post-injection [24]. We detected similar levels of Ad5, Ad5RGE, AdT and AdT*RGE viral transgene expression in MARCO + and Moma-1 + cells (Fig. 6). We failed to detect any co-localisation with MARCO +/Moma-1 + cell in animals pre-treated with clodronate liposomes, as these particular macrophage populations are completely eliminated by the treatment regime ([22] and Supplementary Fig.S2). We detected B220 + transgene-expressing cells extending into the boundaries of the white pulp (WP) and some isolated B220 +/β-Gal + cells were also identified within the centre of the WP (Fig. 7A). We have shown previously that virus transgene expression can be detected within MAdCAM-1 + sinus-lining endothelial cells and ER-TR7 + reticular fibroblasts and fibres [22]. In this study, we found that Ad5 and to a lesser degree, AdT*, co-localised with MAdCAM-1 + endothelial cells (Fig. 7A). Conversely, β-galactosidase expression from RGE-modified vectors, Ad5RGE and AdT*RGE, were detected in close proximity to MAdCAM-1 + cells, but did not co-localise to a significant degree. All vectors appeared to associate to the same degree with ER-TR7 + reticular fibroblasts (Fig. 7A).

We have previously shown that MAdCAM-1 + and ER-TR7 + cells remain unaffected by clodronate pre-treatment ([22] and Supplementary Fig. S2). In macrophage-depleted animals (+ CD), the distribution of transgene expression was largely localised to the MZ, although β-galactosidase + cells were also detected within the white pulp (WP). In agreement with quantitation of genomes, levels of transgene expression in AdT* groups were substantially higher than other treatment groups (~ 3–5 fold higher; p < 0.01).

We detected co-localisation of β-galactosidase with B220 + cells, again at the WP boundaries and within the germinal centres. We also detected AdT* transgene expression in MAdCAM-1 + and ER-TR7 + cells, as previously reported [22]. Similar to untreated (− CD) animals, co-localisation of β-galactosidase with MAdCAM-1 + endothelial cells was limited to Ad5 and AdT*-treated groups and was not detected in Ad5RGE or AdT*RGE groups. All viruses were found to be associated with ER-TR7 + reticular fibroblasts (Fig. 7), though this was more clearly evident for AdT* due to increased amounts of virus in the spleens of these animals.

### 3.6 Altered inflammatory profiles in response to RGE-mutant Ads

**Fig. 8:**
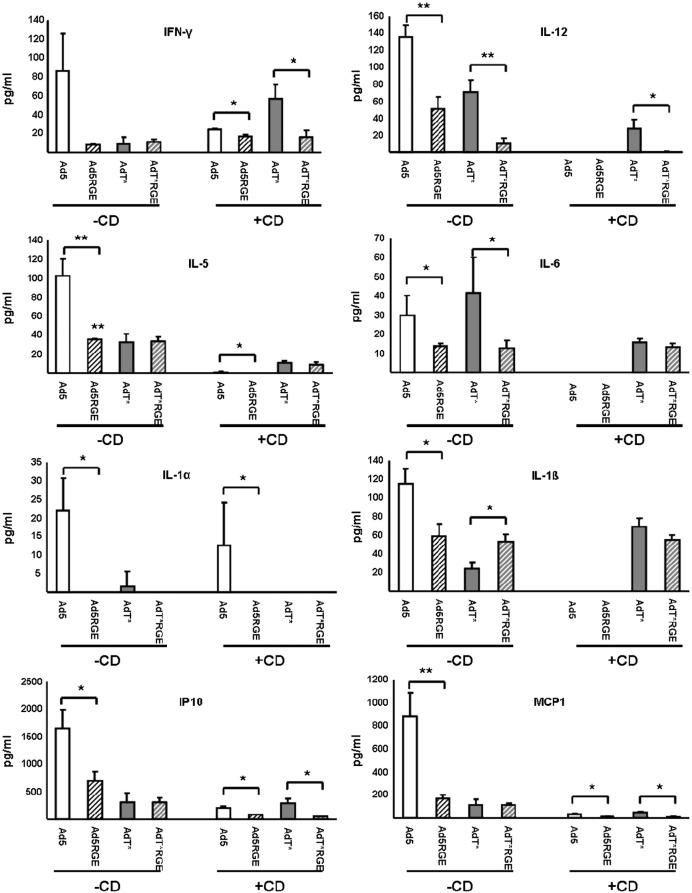
Chemokine/cytokine analysis of sera from mice 6h after administration of RGE-mutant Ads. Chemokines and cytokines in sera from mice 6 h after intravascular administration of 1 x 10^11^ vp Ad5, Ad5RGE, AdT* or AdT*RGE were quantitated using a mouse 20-plex Luminex kit according to manufacturer’s instructions. Values expressed as mean +/- SEM. *=p < 0.05, ** = p < 0.01 compared to parental vector (Ad5 or AdT*).

Several studies have shown that *iv* delivery of Ad5 vectors to naïve mice triggers a robust inflammatory and cellular immune response, with elevated levels of serum cytokines and chemokines observed for several hours after administration [24–27,30,35]. To identify the effect of the penton base RGE mutation on the systemic toxicity associated with *iv* delivery of both control (Ad5) and FX binding-ablated (AdT*) vectors, serum was obtained from all groups of mice 6 h after administration of Ad5, Ad5RGE, AdT* or AdT*RGE (Fig. 8). In non-macrophage-depleted mice (− CD), Ad5 induced significantly higher levels of IL-12, IL-5, IL-1α, IL-1β, IP-10 and MCP-1 than any of the other vectors (p < 0.05 or p < 0.01). Macrophage depletion markedly attenuated this effect, with only interferon-gamma (IFN-γ), IL-5, IL-1α, IP-10 and MCP-1 present at detectable (albeit significantly reduced) levels in Ad5 animals. Conversely, in macrophage-depleted animals AdT* induced significantly higher levels of IFN-γ, IL-12, IL-5, IL-6 and IL-1β than Ad5 vectors (p < 0.05; Fig. 8). Interestingly, mutation of the Ad5 penton base RGD motif significantly attenuated the increase in IL-12, IL-5, IL-6, IL-1α, IL-1β, IP-10 and MCP-1 observed in non-macrophage-depleted Ad5 animals. Similarly, significantly reduced levels of IL-12, IL-6 and IL-1α were observed in non-macrophage-depleted AdT*RGE animals compared to animals receiving the parental AdT* vector. A significant reduction in IL-12, IP-10, IFN-γ and MCP-1 was also observed in macrophage-depleted animals administered AdT*RGE compared to their AdT* counterparts. Taken together, these data suggest that mutation of the penton RGD motif in both the Ad5 and AdT* background significantly attenuates the Ad-mediated induction of selected inflammatory factors after *iv* administration.

## 4 Discussion

The goal of the present study was to generate and characterise penton base RGE-mutated Ad5 vectors on both control and FX binding-ablated backgrounds, in order to assess the contribution of penton base:integrin interactions to the increased spleen uptake and inflammatory responses observed after intravascular administration of FX binding-ablated Ad vectors.

All mutant adenoviruses were successfully grown in HEK293 cells, yielding relatively high particle titers and similar VP/PFU ratios after two-step CsCl ultracentrifugation. Silver stain analysis showed almost identical capsid composition profiles for Ad5RGE and AdT*RGE viruses and their parental Ad5 and AdT* vectors, indicating that single or dual ablation of FX- and RGD-integrin binding had no significant effect on virion assembly and propagation. RGE-mutant adenoviruses also retained the ability to bind and transduce CAR^high^ A549 and CAR^low^ SKOV3 cells in the absence of FX. Although the classical model of Ad5 entry indicates that integrins are required for virus internalisation and endocytosis, studies on the canine adenovirus CAV-2 (which does not possess a penton base RGD motif) have shown co-localisation of CAR with fluorescently-labeled CAV-2 particles in axonal transport vesicles [36]. Ad5RGE and AdT*RGE may therefore use alternate internalisation pathways to gain entry to cells in the absence of a functional penton base RGD motif. In the presence of FX, no increase in AdT* or AdT*RGE cell binding or transduction was observed, consistent with previous studies on the AdT* vector [17]. Similarly, high levels of FX-mediated binding and transduction of A549 and SKOV3 cells by Ad5 and Ad5RGE vectors was observed, in accordance with previous data showing that ablation of the penton base RGD motif does not affect FX-mediated binding and transduction [18]. Importantly, these results indicate that mutation of the penton RGD motif on a FX binding-ablated AdT* background does not significantly compromise the capsid composition or infectivity of this vector.

Assessment of virus biodistribution after *iv* delivery of Ad5, Ad5RGE, AdT* or AdT*RGE to macrophage-depleted and non-macrophage depleted control mice revealed similarly high levels of liver transduction by Ad5 and Ad5RGE, confirming the redundancy of the penton RGD motif for FX-mediated hepatocyte uptake in vivo. Interestingly, a 5-fold reduction in spleen uptake of Ad5RGE compared to the parental Ad5 vector was observed in non-macrophage-depleted animals. Immunohistochemical analysis of spleen sections from these animals showed that although both Ad5 and Ad5RGE transduced marginal zone cell populations, significant transgene colocalisation with MAdCAM-1 + sinus-lining endothelial cells was only observed in Ad5-administered animals. Similarly, a comparable 6-fold reduction in spleen uptake and transduction was accompanied by decreased colocalisation of the β-galactosidase transgene with MAdCAM-1 in spleen sections from macrophage-depleted AdT*RGE animals compared to AdT* counterparts. Previous studies indicate that activation of sinusoidal endothelial cells in the liver after *iv* delivery of Ad5 is significantly attenuated by deletion of the penton base RGD motif [37,38], whilst in vitro transduction of human pulmonary artery, coronary artery and umbilical vein endothelial cells can be significantly increased by insertion of an RGD motif into the Ad5 fibre knob domain [39]. Taken together, these data suggest that uptake of Ad5 vectors by sinus-lining endothelial cells in the spleen may be mediated by an interaction between the penton base RGD motif and cellular integrins. Further detailed in vivo studies on the role of liver and spleen endothelial cells at early timepoints after intravascular delivery of Ad5 are required to confirm this hypothesis. Moreover, as ablation of the penton base RGD motif did not entirely eliminate spleen uptake after intravascular delivery of Ad5 or AdT* vectors, further investigations on the alternate mechanisms underlying transduction of this organ are also essential.

Although a significant component of the anti-Ad5 inflammatory and innate immune response is due to the intracellular recognition of viral DNA and RNA sequences by immune sensors such as the NLRP3 inflammasome [29,40] and cytosolic defensins [41], studies have also identified a role for the capsid penton base protein in RGD integrin-mediated Ad5 uptake and activation of immune cell types, including bone marrow macrophages [30], bone marrow-derived dendritic cells [42] and splenic marginal zone macrophages [24]. RGD-mediated Ad5 uptake by these cells after intravascular delivery is associated with the upregulation of numerous inflammatory cytokines and chemokines, including IFN-γ, IP-10, TNF-α and the MIP-1 proteins [24,30]. Interestingly, transduction of human umbilical vein endothelial cells by a penton base RGD-ablated Ad5 vector is associated with significantly reduced induction of several chemokines, including IP-10 [37]. Di Paolo et al.'s elegant dissection of the IL-1α-mediated, toll-like receptor and inflammasome-independent innate immune response elicited by interactions between Ad5 and cell surface receptors indicate that spleen macrophage populations may be an important source of circulating inflammatory cytokines at early timepoints after intravascular administration of Ad5 [24]. The spleen was also found to be an important site of inflammatory cytokine and chemokine expression in an earlier study investigating early inflammatory activation after *iv* delivery of fibre-mutated Ad5 vectors [43]. In accordance with these studies, the decreased spleen uptake of Ad5RGE compared to Ad5 after intravascular delivery to non-macrophage-depleted animals was accompanied by a significant decrease in the levels of several inflammatory mediators, including IL-12, IL-6, IP-10 and MCP-1. Macrophage depletion also resulted in a general reduction in circulating inflammatory cytokines and chemokines. Interestingly, the increased spleen uptake of AdT* after intravascular administration to macrophage-depleted mice was accompanied by significantly elevated levels of IFN-γ, IL-12, IL-6 and IL-1β, indicating that cells unaffected by clodronate liposome-mediated macrophage depletion may be an important source of cytokines and chemokines in these animals. As stated previously, ablation of the penton RGD motif in AdT* attenuated spleen uptake and reduced levels of circulating inflammatory factors.

Taken together, these results provide valuable information indicating that novel adenoviral vectors with dual mutation of the penton base RGD motif and hexon:FX-interacting residues constitute a viable and attractive platform for intravascular gene therapy applications.
