# Supplementary material for: Biodistribution and inflammatory profiles of novel penton and hexon double-mutant serotype 5 adenoviruses
Source: J Control Release. 2012 Dec 28;164(3):394–402. doi: 10.1016/j.jconrel.2012.05.025 (PMC3520007; doi:10.1016/j.jconrel.2012.05.025)
Supplement: Supplementary material [file mmc1.pdf]

Supplementary figures

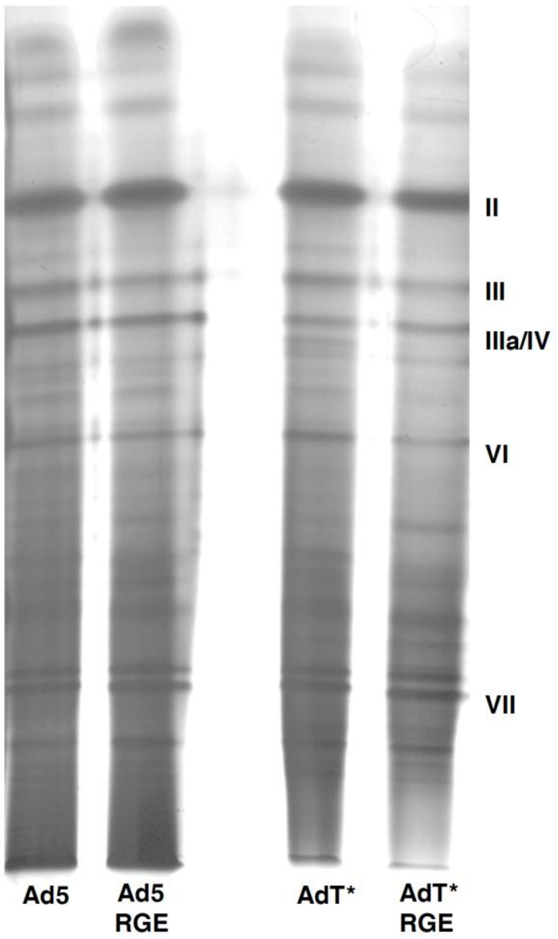

Fig.S1 Adenoviral capsid composition analysed by silver stain. SDS-polyacrylamide gels were loaded with $5x10^{10}$vp of each adenovirus and stained using the PageSilver silver staining kit according to manufacturer's instructions.

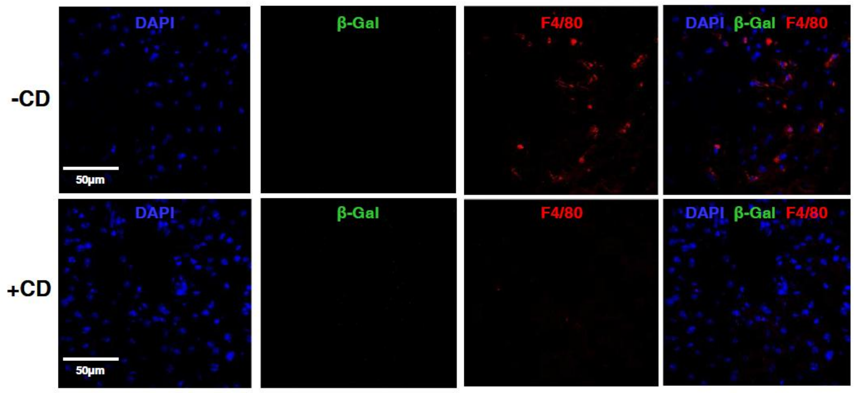

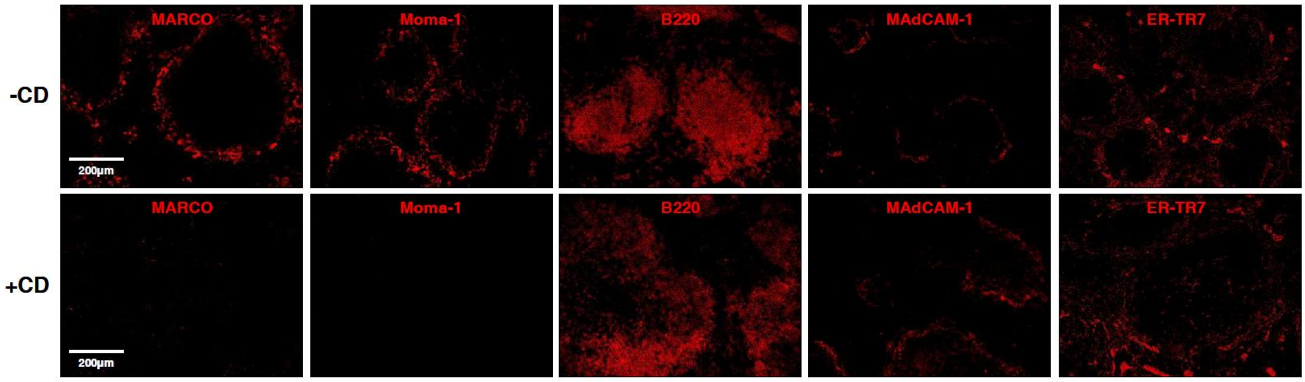

Fig.S2 Selective depletion of macrophage populations after clodronate liposome pretreatment. Immunohistochemical analysis of 4μm frozen liver sections or 6μm frozen spleen sections from PBS-administered, non-macrophage-depleted (-CD) or macrophage-depleted (+CD) mice **Upper panel:** Single and merged images of ß-galactosidase and F4/80 expression in liver sections from control or macrophage-depleted mice 96 hours after intravascular administration of clodronate liposomes. **Lower panel:** Images of MARCO, Moma-1, B220, MAdCAM-1 and ER-TR7 expression in spleen sections from control (-CD) or macrophage-depleted (+CD) mice 96 hours after intravascular administration of clodronate liposomes.
